# Identification of a Primary Stroma and Novel Endothelial Cell Projections in the Developing Human Cornea

**DOI:** 10.1167/iovs.61.6.5

**Published:** 2020-06-03

**Authors:** Eleanor M. Feneck, Philip N. Lewis, Keith M. Meek

**Affiliations:** Structural Biophysics Research Group, School of Optometry and Vision Sciences, Cardiff University, Cardiff, United Kingdom

**Keywords:** cornea, development, elastic fibers, human, cell migration, electron microscopy, primary stroma

## Abstract

**Purpose:**

To investigate the initial events in the development of the human cornea, focusing on cell migration, and extracellular matrix synthesis and organization. To determine whether elastic fibers are present in the extracellular matrix during early human corneal development.

**Methods:**

Human corneas were collected from week 7 to week 17 of development. An elastic fiber-enhancing stain, tannic acid–uranyl acetate, was applied to all tissue. Three-dimensional serial block-face scanning electron microscopy combined with conventional transmission electron microscopy was used to analyze the corneal stroma.

**Results:**

An acellular collagenous primary stroma with an orthogonal arrangement of fibrils was identified in the central cornea from week 7 of corneal development. At week 7.5, mesenchymal cells migrated toward the central cornea and associated with the acellular collagenous matrix. Novel cell extensions from the endothelium were identified. Elastic fibers were found concentrated in the posterior peripheral corneal stroma from week 12 of corneal development.

**Conclusions:**

This study provides novel evidence of an acellular primary stroma in the early development of the embryonic human cornea. Cell extensions exist as part of a communication system and are hypothesized to assist in the migration of the mesenchymal cells and the development of the mature cornea. Elastic fibers identified in early corneal development may play an important role in establishing corneal shape.

The cornea relies on a series of precisely controlled events to develop a stroma that is biomechanically strong but also transparent. Within the avian cornea, development is believed to occur via the induction of cells into the central cornea by an acellular collagenous primary stroma, cells that then go on to secrete the mature corneal stroma.^1^^,^^2^ A primary stroma has not been previously identified in human corneal development, with studies proposing that the human cornea follows similar developmental events to other mammalian developmental models without the presence of a primary stroma.^3^^,^^4^ This has left the events that control mesenchymal cell migration, which in turn leads to the deposition and organization of the extracellular matrix, to remain elusive.

The human cornea initially develops with the surface ectoderm overlying the lens, which eventually detaches to become the corneal epithelium.^5^^,^^6^ After this, there is a migration of mesenchymal-derived neural crest cells that are destined to become the corneal endothelium. Once the endothelium is established, a second wave of mesenchymal cells migrate into the cornea and will form the keratocytes, which will then synthesize and secrete the mature corneal stroma.^7^ The avian cornea proceeds to develop with the same migration pattern of two separate mesenchymal cell migrations.^1^^,^^8^ However, the exact mechanism of how these cells migrate into the presumptive human corneal stroma is elusive compared with the well-investigated avian model, whose mesenchymal cell migrations are initiated and controlled by means of a collagenous primary stroma.^2^

Studying the extracellular matrix composition and organization in the corneal stroma is crucial for determining the developmental events that construct the cornea. Previous work has focused on collagen and proteoglycan deposition and organization within the presumptive corneal stroma, understandably, as these components are crucial to maintain corneal structure and transparency.^2^^,^^9^^–^^12^ The collagen synthesis and secretory pathway have been well investigated within tendon and within avian corneal development and have indicated that collagen is transported from the Golgi apparatus into plasma membranes for deposition.^13^^,^^14^ Specifically, in tendon development, collagen accumulates in plasma membrane fibripositors that align collagen extracellularly in the direction of the fibripositor. Evidence of fibripositors in mammalian corneas has not been found, and the identification of the components responsible for aligning the extracellular matrix, which establishes a transparent cornea, remain to be investigated in the human cornea.^15^ Studies that have analyzed the avian and mouse cornea show that cell processes are responsible for collagen alignment.^15^^,^^16^ In addition, collagen lamellae organization seemed to be associated with the rotation of cell projections, with cell movement before collagen alignment.^17^ These studies highlighted the need to further understand the mechanisms that organize the extracellular matrix in corneal development.

Other than the well-studied collagen and proteoglycan components, elastic fibers have been identified in the posterior peripheral aspect of the mature human cornea and are disrupted in diseases that alter corneal thickness and shape.^18^^-^^20^ These studies demonstrated that elastic fibers are likely to be important for maintaining corneal structure and function, and for establishing corneal structure in embryonic development. To investigate this hypothesis, the present study has analyzed the distribution of elastic fibers from week 7 to week 17 of human corneal development. In most tissues, the development of elastic fibers is essentially complete in embryogenesis, with very little elastic fiber synthesis occurring in mature tissues.^21^ Elastic fibers can exist as true elastic fibers composed of an amorphous elastin core surrounded by fibrillin microfibrils or solely fibrillin-rich microfibrils bundled together.^22^ For the initial assembly of elastic fibers, fibrillin glycoproteins are initially secreted and assemble into fibrillin-rich microfibrils close to the cell surface.^23^ In early development, fibrillin-2 is the main constituent of the fibrillin-rich microfibril system, being replaced by mainly fibrillin-1 with increased maturity.^24^ The fibrillin microfibrils regulate transforming growth factor–β and reinforce the stiffness of tissues.^25^^,^^26^ The fibrillin-rich microfibril bundles can further act as a template for true elastic fiber assembly by depositing onto tropoelastin molecules.^27^^,^^28^ The tropoelastin components undergo coaservation to self-assemble and are further cross-linked by lysyl oxidase to produce mature elastin, which is the core amorphous protein surrounded by fibrillin microfibrils in true elastic fibres.^29^^,^^30^ These true elastic fibers provide elastic properties to skeletal tissues^26^ and could aid the elastic recovery of the cornea.

The aim of this study was to analyze the initial development of the human cornea using novel three-dimensional serial block-face scanning electron microscopy, combined with conventional transmission electron microscopy, to determine whether a primary stroma exists to initiate the migration of mesenchymal cells from the periphery into the central presumptive cornea. The second aim of this study was to further investigate the extracellular matrix components of the fetal cornea, in particular to determine whether elastic fibers are present. Further analysis of the structural distribution of elastic fibers in early corneal development could help determine what role they play, if any, in developing a physiological cornea. Our analysis of the elastic fiber network was focused on the posterior peripheral cornea, because previous studies showed that the elastic fibers predominate in this area of the adult human cornea.^18^

## Methods

### Tissue Collection

All fetal human tissue used in this study was obtained from the Human Developmental Biology Resource (HDBR), Newcastle, UK. The HDBR tissue bank is regulated by the Human Tissue Authority (HTA) and operates under their codes of practice. All tissue samples have been collected with maternal written consent that has been approved by the NRES Committee North East–Newcastle and North Tyneside (REC reference 08/H0906/21+5). All tissue preparation was carried out in accordance with HTA guidelines and the declaration of Helsinki. Corneas were obtained between weeks 7 to 17 and were further dissected into halves, prepared for electron microscopy imaging, and placed in a Karnovsky's fixative for three hours. It is worth noting that, between weeks 7 to 8 the human tissue is embryonic, and the week was further segmented into Carnegie stages CS20 (week 7) and CS22 (week 7.5). After week 8 the tissue is fetal, and ages are determined by the gestation week of the fetus.

### Electron Microscopy

Extracellular matrix and cell contrast was enhanced with a tannic acid–uranyl acetate–based stain, which further enhances the elastic fiber components.^31^ Karnovsky's fixed quadrants were washed in sodium cacodylate buffer three times over 10 minutes and in distilled water (dH₂0) for an additional five minutes. Samples were post-fixed in 1% osmium tetroxide for one hour, and washed with dH₂0 three times over 20 minutes before being transferred to 0.5% filtered tannic acid (TA) in dH₂0 for two hours. Samples were washed with dH₂0 three times over 30 minutes and left overnight in 2% aqueous uranyl acetate (UA). Samples were then dehydrated in a 70% to 100% ethanol series. Samples were further en-bloc stained with 1% UA for two hours, followed by lead acetate in 1:1 ethanol and acetone for two hours. The samples were washed with 1:1 ethanol acetone twice over 20 minutes and then washed three times over 20 minutes with 100% acetone. Samples were infiltrated with 1:1 acetone and araldite resin (araldite monomer CY212 and DDSA hardener) for one hour. BDMA accelerator was added to the premade araldite resin, making continuous resin changes to the samples every two hours until six changes had been made. The samples were embedded and polymerized at 60°C for 48 hours.

#### Serial Block Face-Scanning Electron Microscopy (SBF-SEM)

Samples were placed onto a Gatan specimen pin (Gatan Inc., Pleasanton, CA, USA) and covered with silver conductive epoxy adhesive (TAAB Laboratories, Aldermaston, UK). The pin was sputtered with 7 nm of gold using an ACE200 (Leica Biosystems, Buffalo Grove, IL, USA) and placed inside the Zeiss Sigma VP FEG SEM (Zeiss Group, Oberkochen, Germany) equipped with a Gatan 3View system (Gatan Inc.). The block face surface was imaged on the SEM at 3.5KV at a pixel resolution of 4 nm and a dwell time of 8 µs, and approximately 1000 image slices were acquired from the specimen block every 50 nm by automated serial sectioning. The data sets were recorded at 4096 × 4096 (4K) in Gatan format dm4 files (Gatan Inc.) then batch converted to TIFF format. Three-dimensional reconstructions of the datasets with the isosurface or segmentation functions were composed using Amira 6.4 software (FEI, Mérignac, France).

#### Transmission Electron Microscopy (TEM)

At the end of the serial sectioning, gold ultrathin sections were cut (90 nm) from the SEM 3View sample blocks using the Leica UC6 ultra-microtome (Leica Biosystems). The sections were collected on 300 hexagonal copper grids. The grids were analyzed with the JEOL 1010 transmission electron microscope fitted with a Gatan Orius 1000 TEM camera (Gatan Inc.) at an accelerating voltage of 80KV.

## Results

### Week 7: Carnegie Stage 20

**Figure 1.**
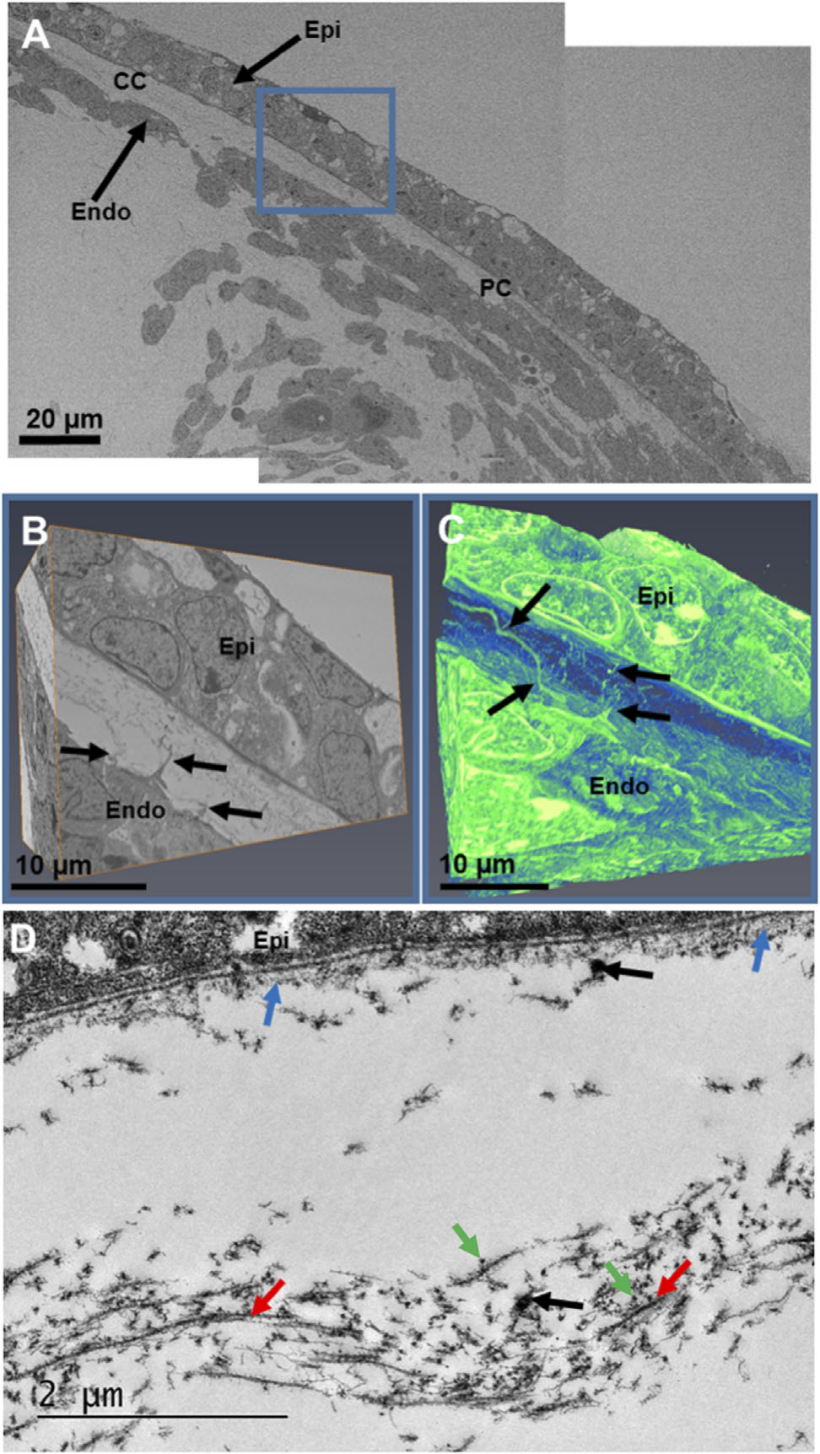
Serial block-face scanning electron microscopy (A–C) and transmission electron microscopy (D) imaging of the embryonic cornea at Carnegie stage 20 during week 7. A low-magnification (×1.14K) stitch of the cornea showed the corneal epithelium (*Epi*) and endothelium (*Endo*), with collagen dispersed between both layers (A). No mesenchymal cells were present in the central cornea (*CC*), with mesenchymal cells confined to the peripheral cornea (*PC*). A high-magnification (×4.27K) dataset was then run for further analysis of the central corneal stroma (*blue box*) (B). Three-dimensional reconstructions of the cornea were rendered into a three-dimensional model using the volren function in the Amira software (C). Three-dimensional reconstructions showed no corneal stromal cells between the corneal epithelium and endothelium, which confirmed that the central cornea was acellular. Cell extensions from the endothelium branched anteriorly and interacted with the basal lamina. In addition, some cell extensions from the epithelium appeared to branch posteriorly. High-resolution images of the presumptive central corneal stroma showed the acellular collagen matrix had condensed (*red arrows*), with collagen fibrils having an orthogonal arrangement to adjacent collagen fibrils (D). The orthogonal arrangement of many collagen fibrils is shown by the longitudinal fibrils (*red arrows*) and the cross-sectional fibrils (*green arrows*). The collagen fibrils were heavily and regularly decorated with proteoglycans. Amorphous extracellular matrix was also seen directly posterior to the corneal epithelium (*blue arrows*). Cell extensions in the corneal stroma (black arrows) correlated with the endothelial cell extensions identified in the three-dimensional models. Refer to Supplementary Video S1 for a movie displaying extra details of the three-dimensional models displayed in C. Green (Cells and cell extensions), blue (Extracellular matrix).

At the start of week 7, serial block-face scanning electron microscopy (SBF-SEM) revealed the presence of the corneal epithelium and endothelium (Fig. 1 and Supplementary Video S1). Between these cellular layers, a collagenous layer had been deposited directly behind the corneal epithelium (Fig. 1D). At the center of the cornea, a condensed collagenous matrix (presumptive corneal stroma) was present, with no mesenchymal cells visible (Figs. 1A and 1D). Three-dimensional models of the cornea reconstructed the epithelium and endothelium and confirmed that no mesenchymal cells had migrated into the presumptive central stroma. The endothelium had cell extensions that branched anteriorly towards the basal lamina and followed a tortuous course. Similar cell extensions branching posteriorly from the epithelium may also be present. These suggested a method of communication between the endothelium and the epithelium. High-resolution TEM images (Fig. 1D) revealed the acellular collagenous matrix to have orthogonally organized collagen fibrils, with an enhancement of collagenous matrix deposition directly posterior to the corneal epithelium and within the central anterior component of the presumptive corneal stroma. The collagen fibrils were heavily and regularly decorated with proteoglycans, as shown in our earlier study.^15^ High-magnification images also revealed cell projections, which confirmed the structures found with SBF-SEM imaging. Refer to Supplementary Video S1 for the video of the three-dimensional model displayed in Figure 1.

### Week 7.5: Carnegie Stage 22

**Figure 2.**
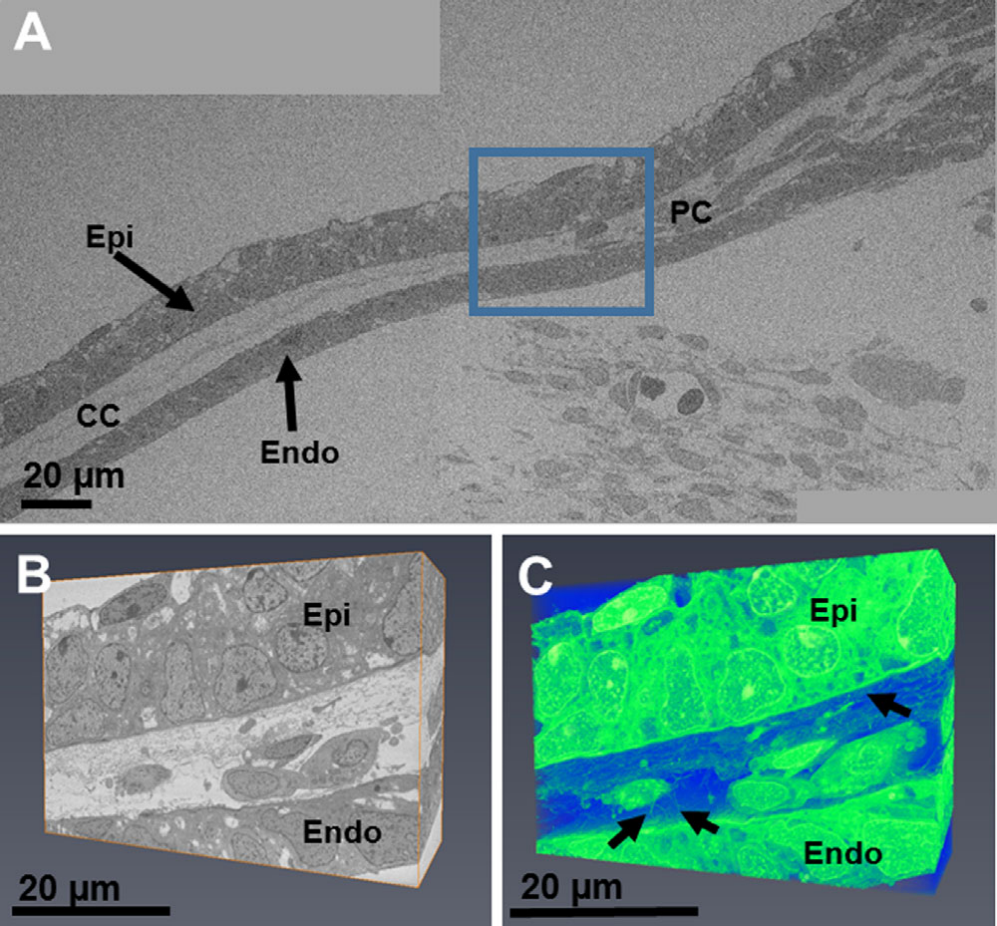
SBF-SEM imaging of the fetal cornea at CS22. A low-magnification (×1.14K) stitch of the developing cornea showed the corneal epithelium (*Epi*) and endothelium (*Endo*), with collagen dispersed between both layers (A). Within the central cornea (*CC*), there were no mesenchymal cells, with just an acellular condensation of collagen fibrils. A high-magnification (×4.27K) dataset from the blue box was then examined for further analysis of the corneal stroma in the peripheral cornea (*PC*) (B). In the peripheral cornea, mesenchymal cells were dispersed between the epithelium and endothelium. Three-dimensional reconstructions of the peripheral cornea showed mesenchymal cells apposed to the collagenous matrix. These cells also contained cell projections to adjacent cells (B and C). Extensions from the corneal endothelium branched toward the collagen matrix and mesenchymal cells (*black arrows*) (C). Please refer to Supplementary Video S2 for increased detail of the three-dimensional model displayed in C. *Green* = cellular material; *blue* = matrix.

**Figure 3.**
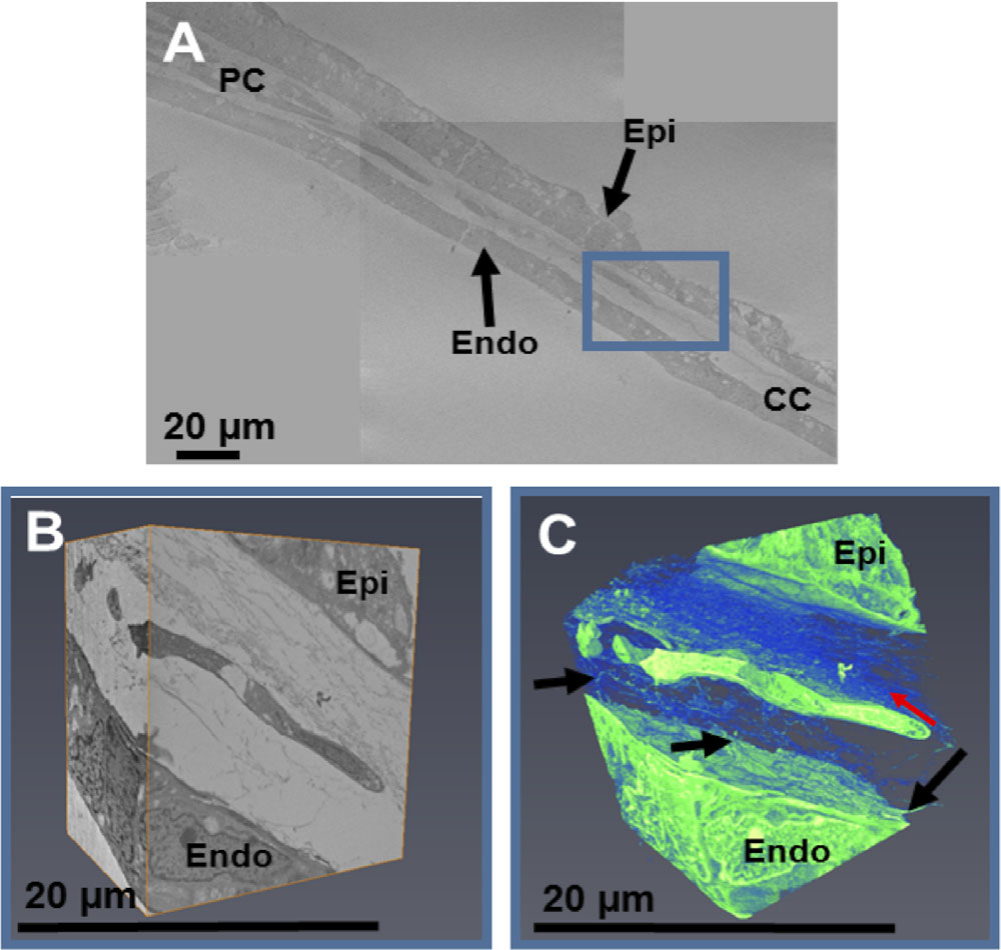
SBF-SEM imaging of the fetal cornea at CS22. The peripheral cornea at CS22 showed individual mesenchymal cells as a monolayer, proximally located when compared to previous ages (A). High-magnification imaging was undertaken of the peripheral corneal cells (*blue box*) (A). Three-dimensional modeling revealed that the mesenchymal cells were close to the condensed collagen fibrils, anterior to the mesenchymal cell (B). Three-dimensional reconstructions of this dataset showed connections from the endothelium (*black arrows*) branching anteriorly toward the mesenchymal cell, with a collagenous matrix lying anterior to the cell (*red arrow*) (C). *Green* = cells and cellular processes; *blue* = extracellular matrix.

**Figure 4.**
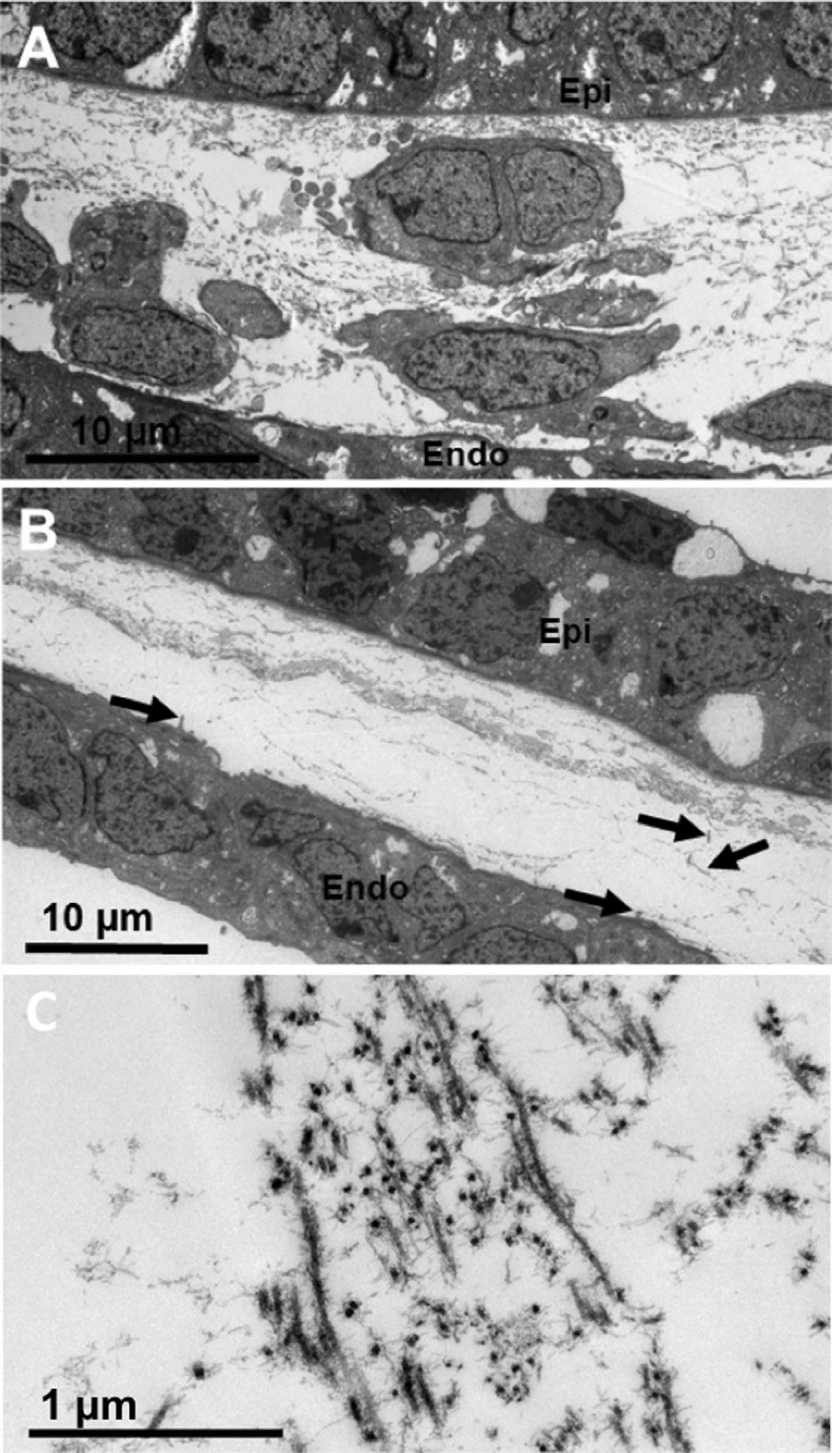
TEM imaging of the developing cornea at CS22. Mesenchymal cells with a rounded morphology were imaged in the peripheral aspect of the developing cornea between the epithelium (*Epi*) and endothelium (*Endo*) (A). Mesenchymal cells were absent from the central corneal stroma, which contained an acellular collagenous matrix condensed within the central anterior region of the presumptive corneal stroma (B). Parts of the cell extensions that project anteriorly from the corneal endothelium towards the acellular collagenous matrix were evident (*black arrows*) (B). The organization of the collagen fibrils in the central corneal stroma have an orthogonal arrangement (C).

At week 7.5, the mesenchymal cells in the periphery of the presumptive cornea seemed to associate with the collagenous layer, with individual cells located closer to the center of the cornea than in the earlier stages (Fig. 2A). Three-dimensional analysis of the peripheral cornea revealed that the cell processes from the endothelium extend anteriorly toward the mesenchymal cells and toward the extracellular matrix and appear to be associated with the migration of mesenchymal cells toward the central cornea (Figs. 2B, 2C). The central region of the corneal stroma remained acellular, with an enhancement of condensed collagen fibrils between the epithelium and the endothelium (Fig. 2A). Refer to Supplementary Video S2 for the full three-dimensional model displayed in Fig. 2C. Further analysis of a more developed cornea at CS22 showed mesenchymal cells forming a monolayer, more centrally located compared with the previous age analyzed (Fig. 3), which indicated the migration of cells into the central cornea. Three-dimensional reconstructions of an individual cell in the peripheral cornea showed that it was embedded within a collagenous matrix, both of which were associated with the corneal endothelial projections that were previously identified (Figs. 3B, 3C). High-resolution TEM images showed mesenchymal cells in the peripheral cornea between the epithelium and endothelium and confirmed the absence of mesenchymal cells within the central cornea (Fig. 4). The central corneal regions contained condensed collagen fibrils, with endothelial cell extensions entering and leaving the plane of the two-dimensional image (Fig. 4).

### Week 8

**Figure 5.**
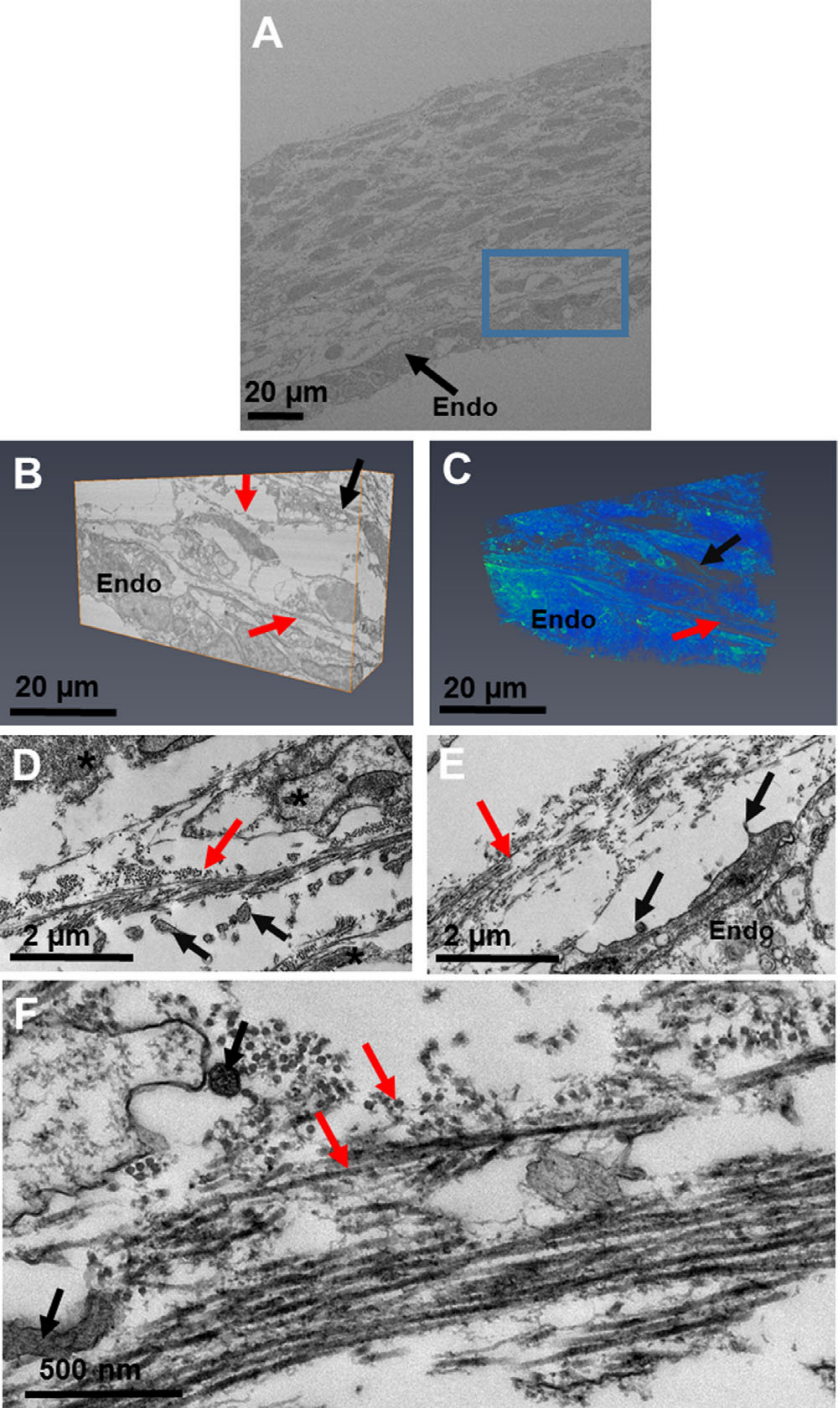
SBF-SEM and TEM imaging of the fetal cornea at week 8. An SBF-SEM image of the central cornea was taken at a low magnification (A). The area of the posterior cornea, directly anterior to the corneal endothelium (*Endo*) was chosen to run a dataset (*blue box*) at a higher magnification (B and C). Three-dimensional models revealed that the corneal stromal cells contain cell projections to adjacent cells (*black arrow*), surrounded by extracellular matrix (*red arrows*). The three-dimensional datasets showed migrated mesenchymal cells distributed throughout the corneal stroma. TEM images (D–F) revealed collagen deposition around the corneal stromal cells in the posterior cornea in better detail. The cells were surrounded by small collagen fibril bundles arranged orthogonally to adjacent collagen fibril bundles (*red arrows*). Cell processes (*black arrows*) were seen, with some extending from the corneal endothelium (E). The corneal epithelium is not present in the images.

By week 8 of fetal development the mesenchymal cells had migrated to populate the entire thickness of the corneal stroma and appeared to have differentiated into corneal stromal cells, which were surrounded by collagen fibrils (Fig. 5). The corneal stroma had dramatically increased in thickness to accommodate the migrated mesenchymal cells (Fig. 5A). The collagen fibrils that surrounded the corneal stromal cells were arranged in small bundles organized orthogonally to adjacent collagen fibril bundles (Fig. 5D). There was no identification of elastic fibers in any datasets analyzed during week 8.

### Week 9

**Figure 6.**
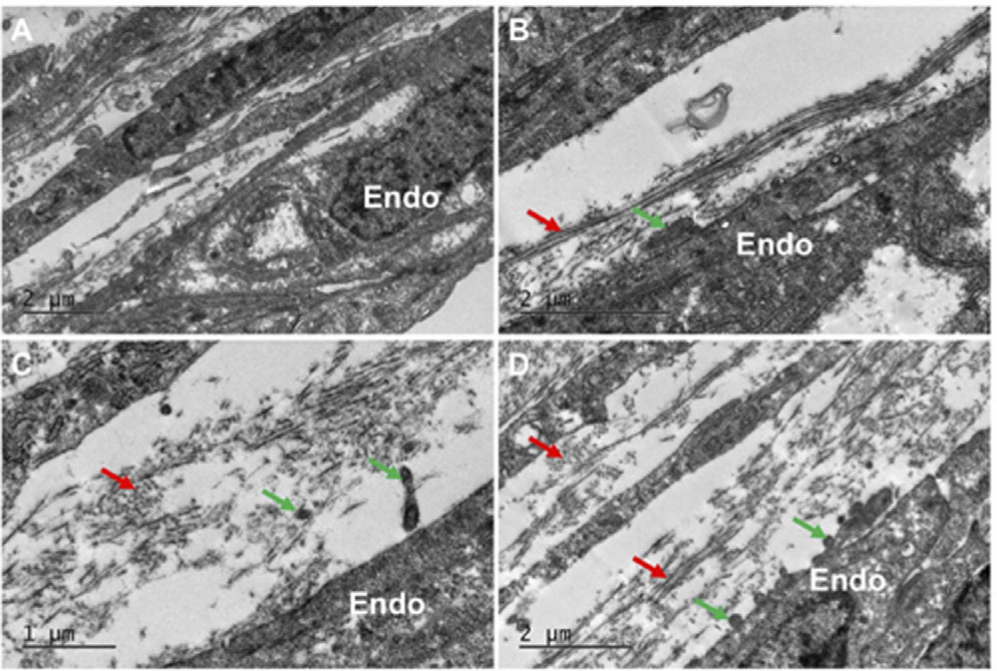
Transmission electron microscopy images of the cornea at week nine of fetal development. In all images within the posterior peripheral cornea, a loose arrangement of collagen fibril bundles could be seen (*red arrows*) (A–D). Cell projections from the corneal endothelium (*green arrows*) extended anteriorly into the corneal stroma and appeared to associate with the collagen matrix within the corneal stroma (B–D). No elastic fibers were observed during week 9 of corneal development.

By week 9 of corneal development, bundles of collagen fibrils were still present. The collagen fibrils were close to the corneal stromal cells and cell membranes (Fig. 6). Cell processes from the corneal endothelium extended into the corneal stromal matrix and associated with collagen. The cells within the corneal stroma possessed a lengthened and flat morphology similar to adult corneal stromal cells. Elastic fibers were not present at week 9 of corneal development.

### Week 12

**Figure 7.**
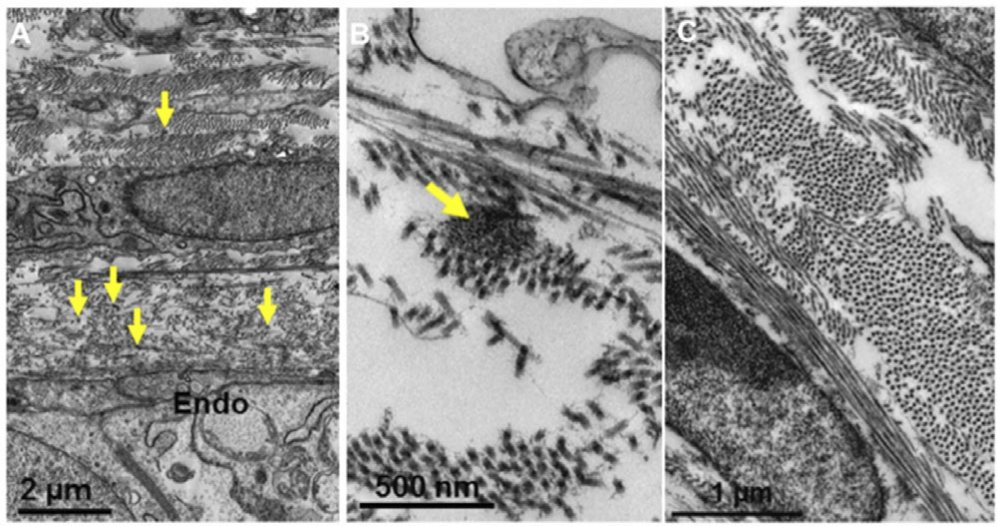
Transmission electron microscopy images of the cornea at week 12 of fetal development. Collagen fibrils were organized into an orthogonal arrangement within the posterior (A and B) and anterior (C) corneal stroma. Heavily stained fibers (*yellow arrows*) were observed directly anterior to the corneal endothelium (*Endo*). These fibers appeared to be composed of bundles of microfibrils when analyzed at a higher magnification, which is typical of elastic fiber morphology (B). No such fibers were found in the anterior or central stroma (C).

By week 12 of corneal development, collagen fibril deposition and organization had increased, with most collagen fibrils organized into orthogonally arranged lamellae (Fig. 7). Interestingly, fibers composed of small rounded clusters of microfibrils were found anterior to the corneal endothelium. These fibers occurred within the posterior peripheral cornea and were absent throughout the rest of the cornea. Cell extensions from the corneal endothelium were not identified from week 12 of corneal development.

### Week 13

**Figure 8.**
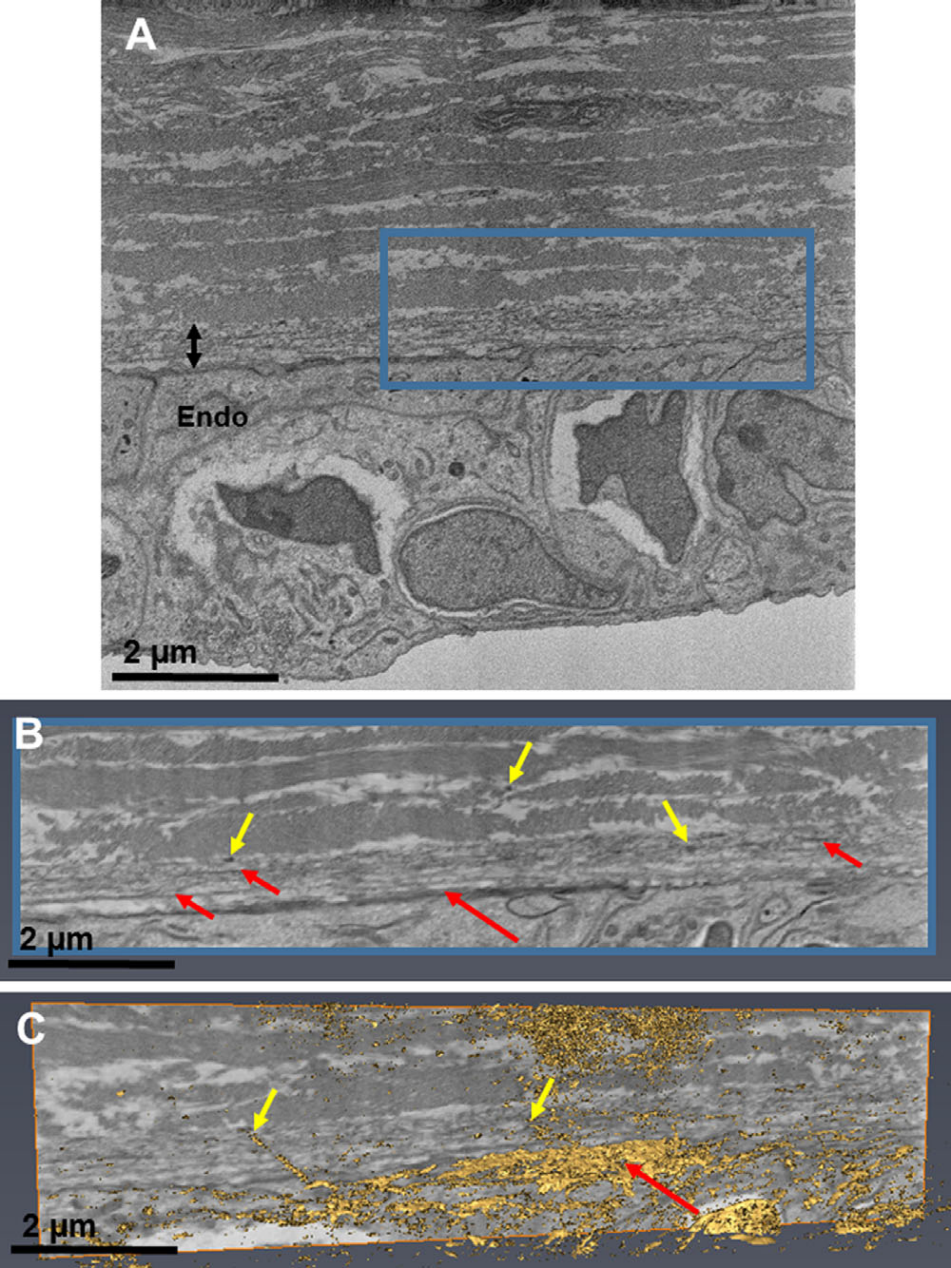
Serial block-face scanning electron microscopy images of the fetal cornea at week 13. The posterior peripheral region of the cornea (A). A high-magnification SBF-SEM data set was run from a region within the *blue rectangle* and three-dimensional reconstructions of the elastic fiber system in an area within this zone were made using the automated isosurface function in Amira (B and^8^ C). These reconstructions identified elastic fibers (rendered in *gold*) concentrated directly anterior to the corneal endothelium, which could represent the initial deposition of Descemet's membrane (*bidirectional arrow* in A; *red arrows* in B and C). Individual elastic fibers (*gold*) were also concentrated in the posterior peripheral region of the corneal stroma (*yellow arrows*).

Collagen deposition and organization continued to increase by week 13, with well-developed collagen lamellae (Fig. 8). Directly anterior to the corneal endothelium, there was a concentration of heavily stained fibers, typical of elastic fibers. The fibers presented as sheets directly anterior to the corneal endothelium with the addition of some individual fibers further within the posterior corneal stroma (Fig. 8). The sheets of elastic fiber material coincide with the position of Descemet's membrane. No elastic fiber-like structures could be identified in the anterior and central corneal regions.

### Week 14-17

**Figure 9.**
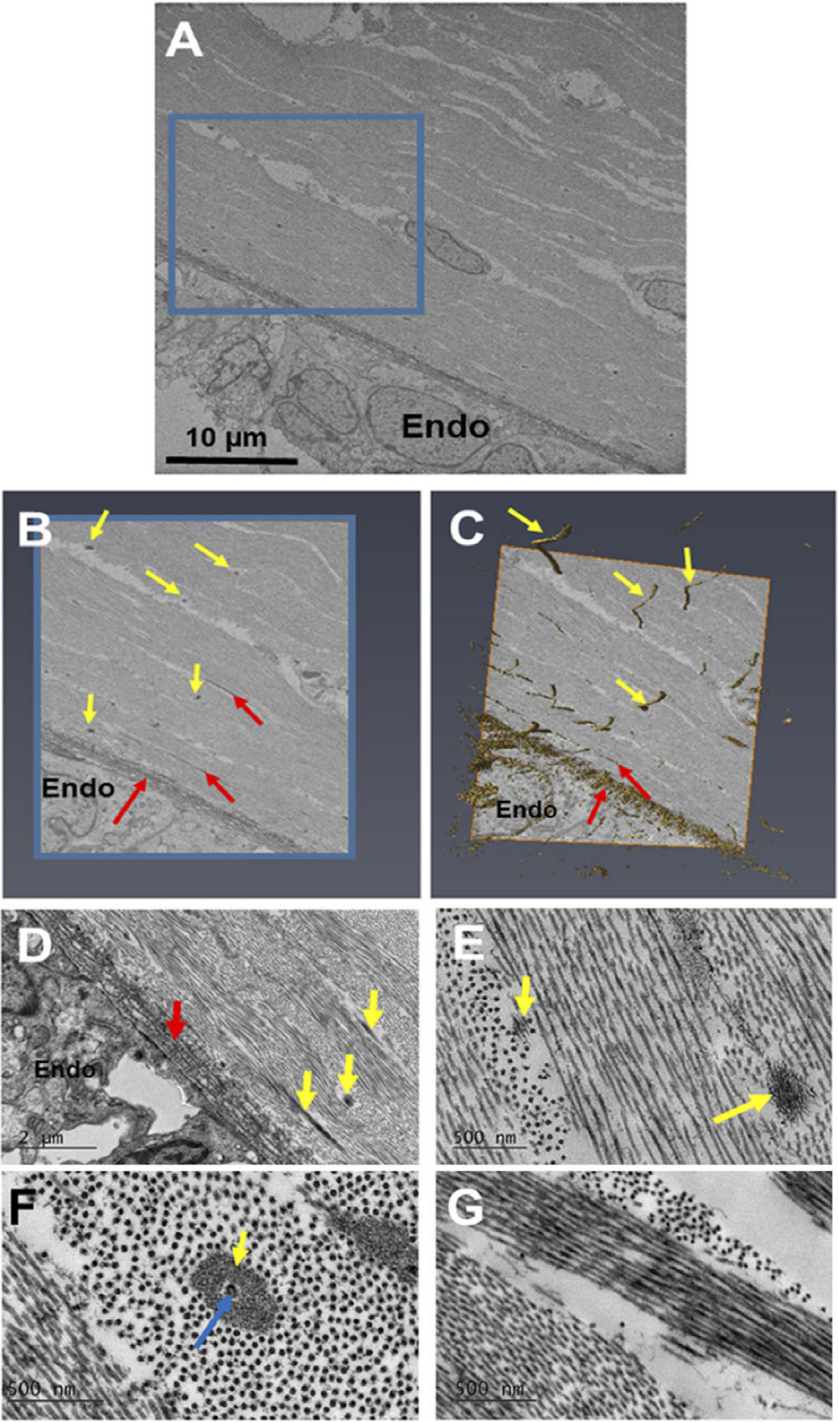
Serial block-face scanning electron microscopy (SBF-SEM) and TEM images of the fetal cornea. The posterior peripheral region of the stroma was chosen for analysis with SBF-SEM of the week 17 fetal cornea (*blue box*) (A–C). B and C are data from within the 3-D region defined by the blue box in A. Elastic fiber sheets were concentrated above the corneal endothelium (*Endo*) (*red arrows*) (A and B). Three-dimensional models showed individual elastic fibers (*gold label*) within the posterior peripheral cornea (*yellow arrow*) (A–D). High-resolution images with TEM showed the elastic fiber sheet directly anterior to the corneal endothelium at week 17 (*red arrows*) (D). Individual elastic fibers were concentrated to the posterior aspect of the corneal stroma, composed of bundles of loosely arranged microfibrils approximately 10–12 nm in diameter (D and E). Some fibers appeared to contain an amorphous core surrounded by bundles of microfibrils, these fibers appeared to represent true elastic fibers (F). No elastic fibers were identified in the anterior or central stroma between weeks 14 to 17; within this area collagen fibrils were organized within lamellae with an orthogonal arrangement between corneal stromal cells (F).

As development progressed, collagen fibrils and lamellae became further organized. Fibers with a similar structure to elastic fibers were imaged in the posterior peripheral cornea. The fibers were directly anterior to the corneal endothelium as sheets and individual fibers within the posterior corneal stroma and appeared to increase in size and density with maturation (Fig. 9). The fibers consisted of clusters of smaller microfibrils ∼10 to 12 nm in diameter, typical of fibrillin-rich microfibrils. In addition, some fibers were found to contain an amorphous core with greater contrast, which is believed to be elastin; these fibers possess a structure similar to true elastic fibers. There was no identification of elastic fibers elsewhere within the corneal stroma.

## Discussion

This study has used three-dimensional imaging techniques to investigate the initial development of the human cornea. We have provided novel evidence of a primary stroma within the early development of the human cornea. In addition, we provide new evidence of three-dimensional endothelial cell projections that associate with the surface ectoderm, collagenous matrix and with the migrating mesenchymal cells located in the peripheral cornea. Our research further indicated that the endothelium has an important communicatory role in human corneal development between weeks 7 and 12. Once the mesenchymal cells had populated the cornea and collagen deposition began, collagen fibrils formed bundles orthogonally organized with respect to adjacent collagen bundles. The collagen fibril bundles associated with cell membranes, and later formed orthogonally arranged collagen lamellae whose organization increased with maturation. The association of collagen fibrils with cell membranes is not a novel finding in the process of collagen fibrillogenesis, and is well described in the avian cornea and within tendon developmental biology.^13^^,^^14^ The final aspect of this study used a tannic-acid uranyl-acetate–based stain to enhance the visualization of elastic fibers within the human corneal stroma. This technique is believed to be specific for elastic fiber enhancement, and this was confirmed in our previous publication using antibody staining specific to elastin and fibrillin-1.^32^ Our results showed that elastic fibers are present from week 12 of fetal development and are confined to the posterior corneal stroma.

From week 7 of corneal development, the corneal epithelium and endothelium are well developed, with no mesenchymal cells between the two cell layers. Instead, an acellular collagenous matrix is present with orthogonally arranged collagen fibrils. Before this study, it was hypothesized that the human cornea followed a similar line of development to other mammalian cornea models, where no acellular primary stroma is present.^3^^,^^4^ This absence of an acellular collagenous matrix is different from the well-studied avian species, where a primary stroma assists the migration of the mesenchymal cells that are responsible for synthesis of the mature cornea.^2^^,^^33^ Our results show that the human embryonic cornea follows a mechanism of development more similar to the avian cornea than previously thought, with the observation of an acellular collagenous matrix orthogonally organized before mesenchymal cell migration, similar to the events that are demonstrated in the avian developing cornea.^2^

Three-dimensional reconstructions of the human cornea at week 7 identified endothelial cell projections that branch anteriorly toward the corneal epithelium, suggesting a communication between both cell types. Interestingly, the endothelial cell projections did not penetrate through the basal lamina; therefore, if these cells are communicating, it is most probably through signaling or mechano-transduction methods, not through direct cell/cell contact methods. With increased development, the collagenous primary stroma condensed with increased density toward the presumptive central anterior stroma, with mesenchymal cells in the peripheral cornea apposed to the collagenous matrix. Three-dimensional models also showed cell projections from the endothelium to branch toward the collagen matrix and mesenchymal cells. These endothelial cell extensions are hypothesized to aid mesenchymal cell migration.

Interestingly, the endothelial cell projections that branched into the corneal stroma were not identified after week 12 of fetal development. We propose that the endothelial cells provide the main communication system within the cornea before mesenchymal cell migration and assist mesenchymal cell migration into the presumptive cornea. We also demonstrate that once the mesenchymal cells have migrated, there is no requirement for the endothelial cells to communicate with the mesenchymal cells, therefore these cell projections are diminished and the corneal stromal cells then provide the main communication with the cell projections they generate to adjacent corneal stromal cells. Further studies should investigate the role of the endothelium and any underlying mechanisms that contribute to the successful development of the cornea.

**Figure 10.**
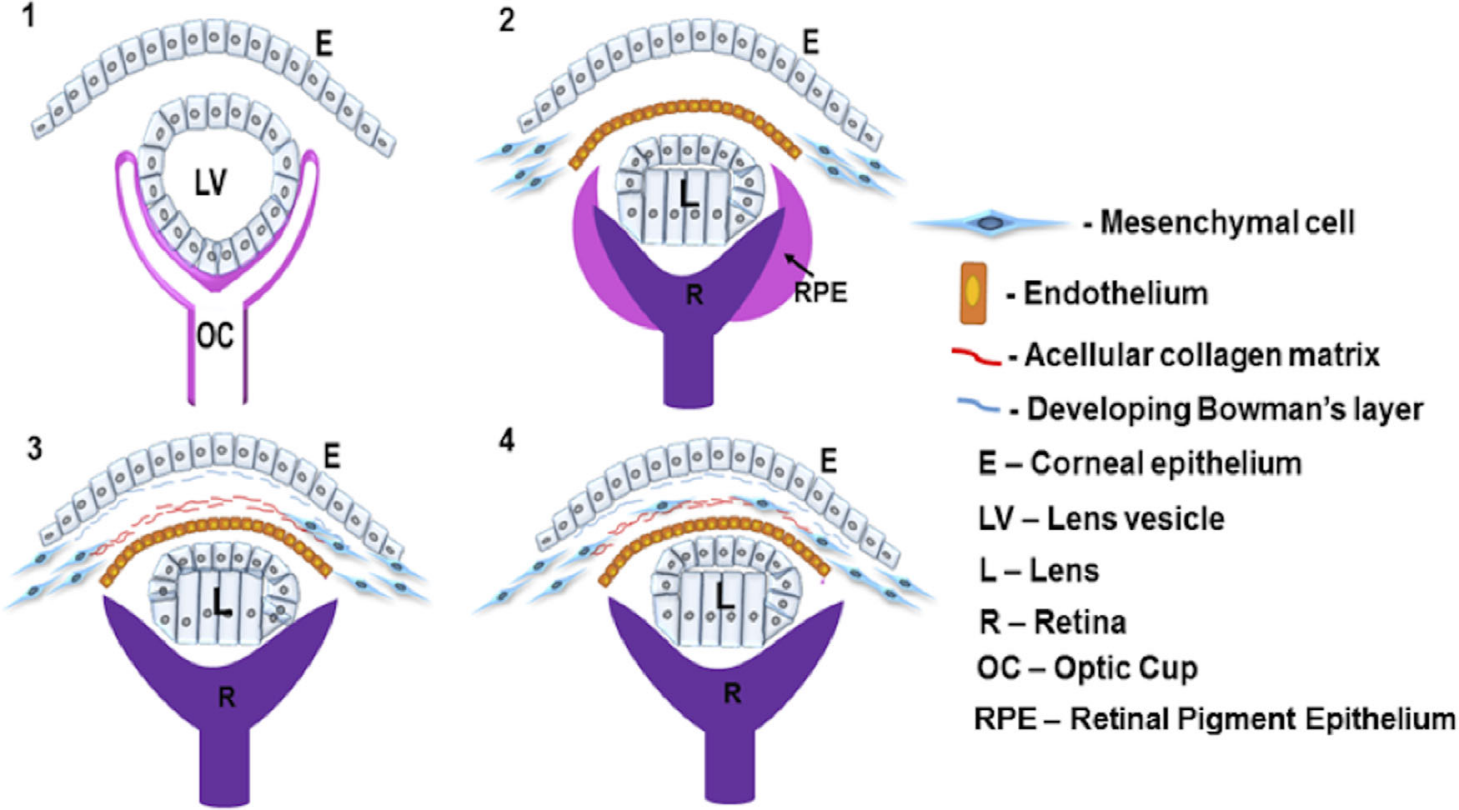
Human corneal development hypothesis. (1) The surface ectoderm detaches away from the lens vesicle and the surface ectoderm becomes the corneal epithelium. A space lies between these structures. (2) The first mesenchymal cell migration proceeds to form the endothelium, anterior to the lens. Mesenchymal cells are present in the periphery of the cornea but not within the central cornea. (3) Collagen fibrils are deposited posterior to the corneal epithelium and within the central cornea. The collagen fibrils in the central cornea condense and align with the cells in the periphery of the developing cornea. (4) The mesenchymal cells use the acellular collagen matrix as a substrate for a second cell migration into the corneal stroma.

**Figure 11.**
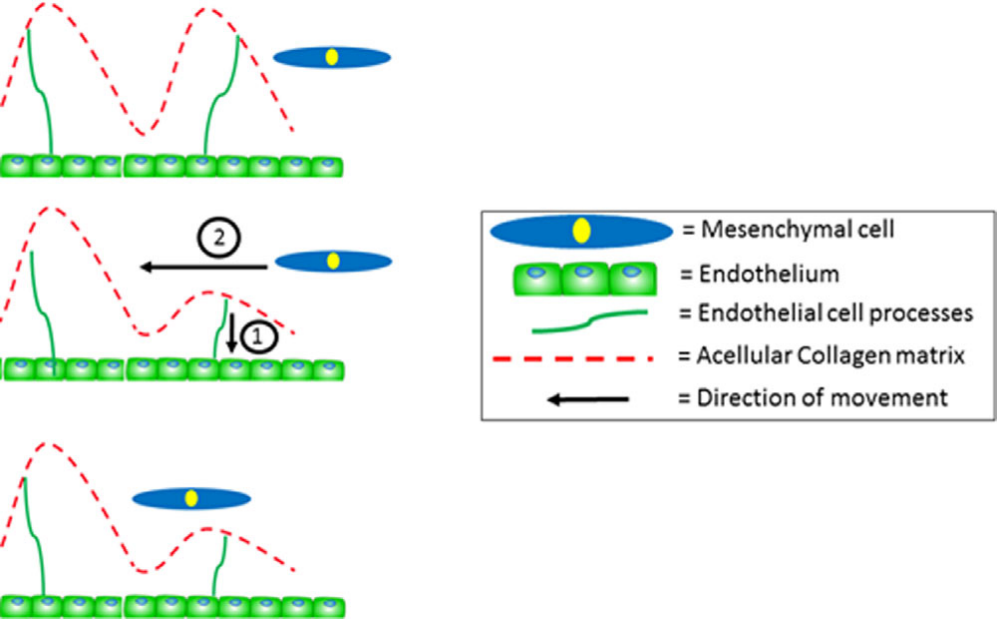
Theory of mesenchymal cell migration. The acellular collagen network sits in the central aspect of the cornea, adjacent to the mesenchymal cells in the peripheral cornea. Mesenchymal cells from the peripheral cornea appear to migrate one cell at a time. We hypothesize that the endothelial cell extensions communicate with the acellular collagen matrix which moves and initiates the cells in the peripheral cornea to migrate to their desired location. This figure shows the endothelial cell extensions moving down (1), which causes the cell matrix to move for the mesenchymal cell to migrate inwards along the collagen matrix (2). The endothelial cell processes would continue to move the collagen matrix and communicate with the mesenchymal cells, until all mesenchymal cells have infiltrated the cornea, reached their desired location and differentiated into corneal stromal cells.

Cells in embryogenesis are well described in many tissues to use neighboring cells, as well as extracellular matrix components as a substrate for cell migration.^34^ Cells can use contact guidance mechanisms within the extracellular matrix to support their migration. We suggest that mesenchymal cells migrate along a collagenous matrix in the human cornea, similar to the mechanism that was previously described in the avian cornea.^1^^,^^2^ Our model of how the human cornea develops is summarized in Figure 10. We also propose that the endothelial cell projections have a role in cell migration, and this theory is further summarized in Figure 11. Future studies should be designed to test the hypothesis of the mechanisms that regulate the events that construct the cornea.

To establish a transparent and biomechanically strong cornea, collagen fibrils with regular diameters must assemble within organized lamellae. There remains much debate on the process of how collagen organization within the human cornea is established. The collagen secretory pathway is well described in tendon development, with collagen transported from the Golgi apparatus to plasma membrane fibripositors, which subsequently deposit and align collagen in a given direction.^13^ Even though it has been recorded that fibripositors have an involvement in avian corneal development, they have not been seen within the mouse cornea.^14^^,^^15^ Further studies should identify whether fibripositors exist within the human cornea, which would further our understanding of the mechanisms that control collagen fibril deposition and organization. In addition, studies should determine the types of collagen and extracellular matrix components that exist within the human stroma before and after mesenchymal cell migration to help identify the mechanisms that proceed cell migration to establish physiological corneal structure and function.

The second aim of this article was to determine whether elastic fibers are present in human corneal development. The tannic acid–uranyl acetate method has been previously described in numerous studies to enhance the contrast for elastic fibers within the cornea.^18^^-^^20^^,^^31^^,^^32^ The elastic fibers were imaged as bundles of fibrillin-rich microfibrils, with some fibers containing an elastin core, characteristic of true elastic fibers, as described by previous studies.^18^ The elastic fibers were initially identified from week 12 of corneal development as small and round bundles of microfibrils concentrated within the posterior peripheral region of the cornea. The elastic fibers showed enhanced staining with increased development from weeks 12 to 17, the final age analyzed in this study. They had a similar distribution to the elastic fibers that have been previously identified in the adult human cornea, being concentrated directly anterior to the corneal endothelium and containing small individual fibers confined to the posterior peripheral corneal stroma.^18^

Additional ultrastructural changes occur after eyelid opening, with maturity being reached approximately six months after birth; therefore we expect the elastic fiber system to keep developing with increased maturity.^35^ Even though the structural distribution of the elastic fibers has been well studied, a functional understanding still remains unknown. Previous studies have shown that conditions leading to changes in corneal shape are associated with a disrupted elastic fiber network.^19^^,^^20^ This led to the theory that elastic fibers maintain corneal curvature and allow the peripheral posterior cornea to deform and return to its physiological shape after being subjected to external forces. The results in this study indicate that elastogenesis occurs in corneal development and therefore could be important to the development of a functional cornea. It should be noted that Bowman's membrane and Descemet's membrane had not begun to form in the stages analyzed in this study; further study should analyze these structures through corneal development.

Our results have demonstrated that human corneal development exhibits some surprising similarities and differences to other species. We showed that the human cornea has the same cellular migration mechanism as the avian cornea, with the presence of a primary stroma.^2^ On the other hand, the human cornea develops with a different mechanism when compared with other mammalian species, including the mouse, where no primary stroma is seen and the cells migrate in a different pattern.^15^ It has yet to be determined whether the cell projections identified in this study are found in developing chick or mouse models.

To conclude, this study has provided strong evidence of an acellular collagenous primary stroma that is present prior to mesenchymal cell migration in the human cornea at week 7. We have also identified that the corneal endothelium contains many cell projections that are associated with the corneal epithelium, collagenous matrix and migrating mesenchymal cells in the very early stages of corneal development. Many of these initial developmental events in the human cornea are more similar to those occurring within the developing avian cornea than previously thought.

## Supplementary Material

Supplement 1

Supplement 2

## Supplementary Material

**Supplementary Video S1**. SBF-SEM three-dimensional reconstruction of the fetal cornea at Carnegie stage 20 during week 7.

**Supplementary Video S2**. SBF-SEM three-dimensional model of the fetal cornea at Carnegie stage 22 during week 7.5.
